# Pain and overall quality of life in palliatively treated colorectal cancer patients 1 year after diagnosis– results from the EDIUM cohort

**DOI:** 10.1007/s00432-025-06186-x

**Published:** 2025-03-31

**Authors:** Sophie Klara Schellack, Clara Breidenbach, Christoph Kowalski, Ulrich Wedding, Birgitt van Oorschot, Thomas Seufferlein, Stefan Benz, Martin Schnell, Jörg Köninger, Christina Klein, Johann Ockenga, Björn Freitag, Uwe A. Wittel, Roger Wahba, Mia Kim, Saleem Elhabash, Pompiliu Piso, Dirk Weyhe, Jörg Bunse, Maren Riechmann, Marco von Strauss, Sebastian Petzoldt, Philipp-Alexander Neumann, Vanessa Kolb, Nora Tabea Sibert

**Affiliations:** 1 German Cancer Society, Kuno-Fischer-Straße 8, 14057 Berlin, Germany; 2 University Medicine Jena, Kastanienstraße 1, 07747 Jena, Germany; 3 University of Würzburg, Josef-Schneider-Straße 11, 97080 Würzburg, Germany; 4 University Medicine Ulm, Albert-Einstein-Allee 23, 89081 Ulm, Germany; 5 Klinikum Sindelfingen-Böblingen, Calwer Straße 68, 71034 Böblingen, Germany; 6 Hegau-Bodensee-Klinikum Singen, Virchowstr. 10, 78224 Singen, Germany; 7 Klinikum Stuttgart, Kriegsbergstraße 60, 70174 Stuttgart, Germany; 8 Helios Park-Klinikum Leipzig, Strümpellstraße 41, 04289 Leipzig, Germany; 9 Bremen Klinikum Bremen-Mitte, Sankt-Jürgen-Str. 1, 28205 Bremen, Germany; 10 St. Josef-Hospital, Gudrunstraße 56, 44791 Bochum, Germany; 11 Kliniken Nordoberpfalz– Klinikum Weiden, Söllnerstraße 16, 92637 Weiden in der Oberpfalz, Germany; 12 Helios Klinikum Berlin-Buch, Schwanebecker Chaussee 50, 13125 Berlin, Germany; 13 München Klinik Neuperlach, Sanatoriumspl. 2, 81545 München, Germany; 14 Johannes Wesling Klinikum Minden, Hans-Nolte-Straße 1, 32429 Minden, Germany; 15 Barmherzige Brüder Regensburg, Prüfeninger Str. 86, 93049 Regensburg, Germany; 16 Pius Hospital University Medicine Oldenburg, Georgstraße 12, 26121 Oldenburg, Germany; 17 Sana Klinikum Lichtenberg, Fanningerstraße 32, 10365 Berlin-Lichtenberg, Germany; 18 Sana Klinikum Hof, Hochfranken, Eppenreuther Straße 9, 95032 Hof, Germany; 19 St. Claraspital Basel, Kleinriehenstrasse 30, Basel, 4058 Switzerland; 20 DRK Kliniken Berlin-Treptow– Köpenick, Salvador-Allende-Str. 2– 8, 12559 Berlin, Germany; 21 Klinikum rechts der Isar, TUM University Hospital, TU Munich, Ismaninger Str. 22, Munich, Germany; 22 OnkoZert GmbH, Gartenstraße 24, Neu-Ulm, Germany; 23 Oncological Health Services Research with a focus on Digital Medicine, Department of Gynaecology and Obstetrics, CIO ABCD, University Hospital Düsseldorf, Heinrich-Heine University Düsseldorf, Moorenstraße 5, Düsseldorf, Germany

**Keywords:** Palliative care, Colorectal neoplasms, Patient reported outcomes, Pain, Quality of life

## Abstract

**Purpose:**

Diagnosis with UICC stage IV colorectal cancer often indicates palliative treatment to alleviate symptoms. Data on pain in these patients are still scarce but can help improve symptom management. This study therefore aimed to describe patient-reported pain and quality of life.

**Methods:**

147 palliatively treated stage IV colorectal cancer patients diagnosed between 2018 and 2023 completed the EORTC QLQ-C30 and QLQ-CR29 before and 12 months after treatment initiation within the EDIUM study. Descriptive results for pain and quality of life were examined and compared to reference values. A logistic regression analysis investigated the relationship between quality of life and pain and 1-year survival.

**Results:**

The mean (SD) for the “overall pain” score was 26 (32) (T0) and 35 (32) (T1) for rectal cancer patients and 34 (33) (T0) and 35 (32) (T1) for colon cancer patients. This is higher than the reference value (24 (30)) and indicates high average pain levels. The “overall quality of life” score showed means below the reference value (61 (23)), indicating poorer quality of life (colon: 51 (25) (T0), 56 (22) (T1); rectum: 52 (24) (T0), 51 (22) (T1)). Higher pain levels persisted at both time points. The logistic regression results suggest a small relationship between pain and quality of life and 1-year survival.

**Discussion:**

This study reveals high levels of pain among palliatively treated colorectal cancer patients, impacting their quality of life. Effective pain management and close monitoring are necessary to improve the quality of life for these patients.

**Trail number:**

DRKS00008724.

**Supplementary Information:**

The online version contains supplementary material available at 10.1007/s00432-025-06186-x.

## Background

Colorectal cancer (ICD-10 C18-C20) is one of the most common cancers in Germany. Approximately 55,000 new cases are diagnosed each year in Germany, with approximately 24% of the female and 26% of the male patients being diagnosed with UICC stage IV, indicating distant metastases of the primary tumour (Robert Koch-Institut 2023).

Clinical guidelines for metastatic cancer define prolongation of survival, alleviation of the symptoms, and improvement of quality of life as the primary aims when complete surgical resection of the tumour cannot be achieved. In these cases, palliative care– either with or without tumour-directed therapy– is often the preferred choice of treatment (German Guideline Program in Oncology 2019; Sanders et al. 2024). The oncological S3 guideline for palliative care sets out the principles of care for patients with incurable cancer, including a symptom-specific treatment approach (German Guideline Programme in Oncology 2015).

For cancer patients, pain is a major concern, and dealing with pain can interfere with daily activities and limit quality of life (Kenzik et al. 2015; Rodriguez et al. 2019). Patient-reported outcomes (PROs) are important measures for assessing outcomes such as pain. PROs are outcomes reported by patients themselves, using validated paper or online questionnaires. They measure the subjective status of symptoms and functions before, after, or during a therapeutic intervention, can serve as a complement to therapeutic success, and are therefore increasingly important for medical care (Di Maio et al. 2022). Since one goal of care defined in the oncologic guidelines for palliatively treated patients is to improve patients’ quality of life, the assessment of PROs is the gold standard for adequately managing symptoms. Studies report heterogeneous frequencies of colorectal cancer patients experiencing pain, with a lack of studies that focus particularly on pain symptoms in palliative colorectal cancer patients (Drury et al. 2017; Zielińska et al. 2021).

This study aims to describe pain in palliatively treated stage IV colorectal cancer patients at baseline and 1 year after diagnosis.

## Methods

### EDIUM study

The EDIUM study (*“*Outcome Quality in Colorectal Cancer: Identification of Differences and Measures for Nationwide Quality Development”) is an ongoing multicentre prospective observational study in Colorectal Cancer Centres that are certified in accordance with the requirements of the German Cancer Society, with the goal of comparing the quality of care for colorectal cancer patients between centres. Study data include functional and symptomatic outcomes as part of the PRO questionnaires used in the study, and clinical end points based on quality assurance data reported as part of the certification process. Currently, data from more than a hundred Colorectal Cancer Centres enrolling their patients in the EDIUM study are available. Details of the EDIUM study are available elsewhere (Kowalski et al. 2022).

### Study population

This subgroup analysis focused on palliatively treated stage IV colorectal cancer patients in Germany. The study population consists of colorectal cancer patients treated in Colorectal Cancer Centres enrolled in the EDIUM study. Patients included in the EDIUM study are asked to complete a baseline questionnaire prior to the initiation of any treatment (T0) and another questionnaire 12 months after the start of treatment (T1). The questionnaires include sociodemographic questions as well as the European Organisation for Research and Treatment of Cancer (EORTC) quality of life questionnaire EORTC QLQ-C30 and the EORTC QLQ-CR29 (Giesinger et al. 2020; Whistance et al. 2009). The assessment time points (T0, T1) as well as the set of PROs for colorectal cancer patients were chosen based on the ICHOM recommendations and the EORTC Manual for the use of EORTC measures in daily clinical practice (Wintner et al. 2016; Zerillo et al. 2017).

**Fig. 1:**
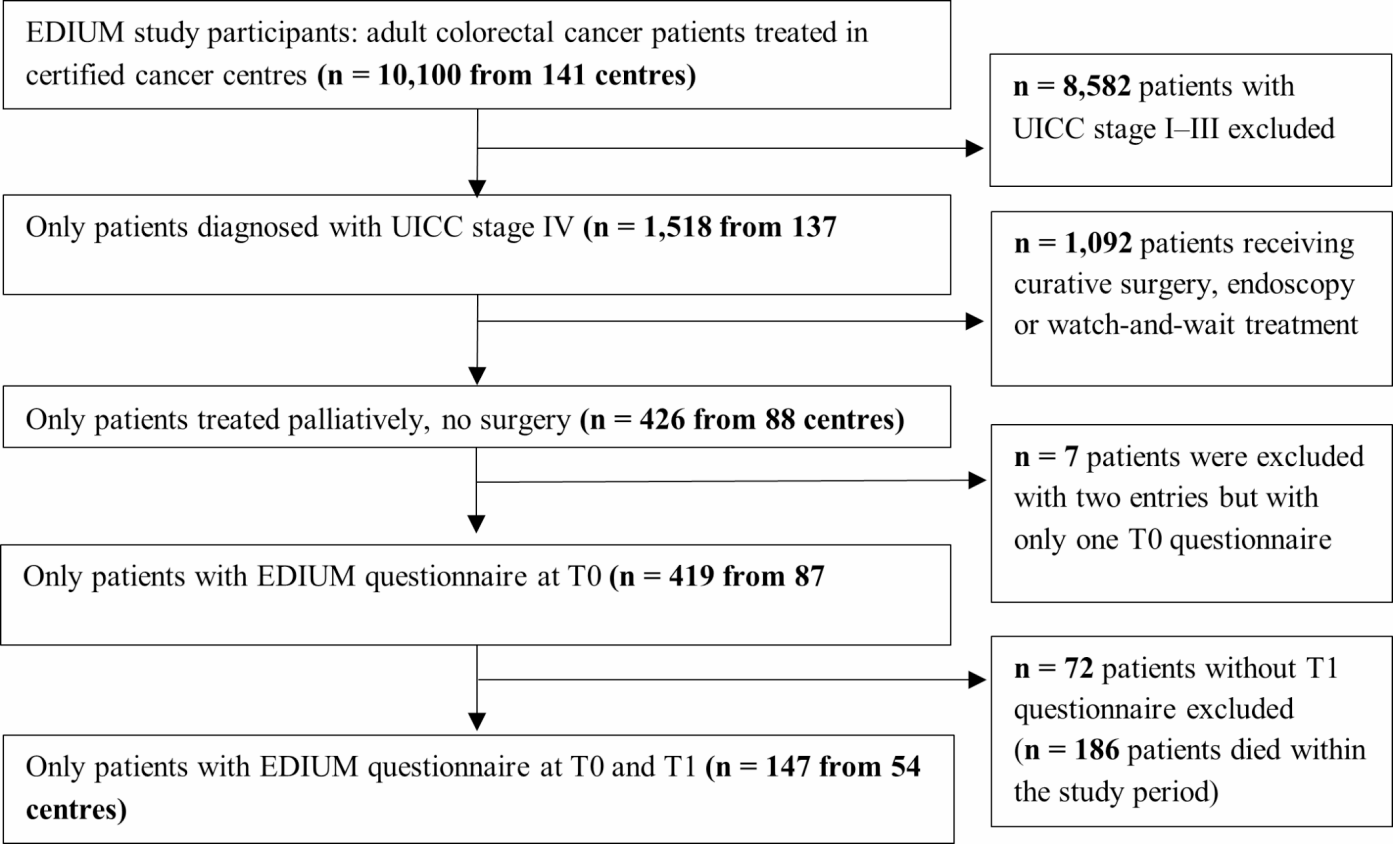
Consolidated Standards of Reporting Trials (CONSORT) chart for the EDIUM study sample

Only palliatively treated patients in stage IV without tumour resection who had completed both questionnaires at T0 and T1 were analysed in this study (Fig. 1). Additionally, patients who did not fill out T1 or died within the 12-month period before it were analysed separately.

### Outcomes

To address pain in palliatively treated colorectal cancer patients, both the total pain score from the C30 and the colorectal cancer–specific pain scores, as well as the corresponding single items were examined. The C30 “pain” score consists of two items (9: “pain” and 19: “Interference with daily activities due to pain”). The response scale for all pain items comprises “not at all”, “a little”, and “quite a bit” to “very much” and is converted into score values ranging between 0 and 100, with higher values indicating more severe pain (Aaronson et al. 1993). The CR29 colorectal cancer–specific pain items address “dysuria” (34), “abdominal pain” (35), and “buttock pain” (36) during the previous week. These items are also converted into scores ranging between 0 and 100, with higher values indicating more severe pain. In addition, overall quality of life is assessed, which also consists of two items (29: “global health status” and 30: “overall quality of life), ranging between 0 and 100, with a higher value indicating a better status (Whistance et al. 2009). The calculation of the raw score and the linear transformation of the items was applied according to the EORTC QLQ-C30 Scoring Manual (Fayers et al. 2001).

To identify clinically relevant changes in function and symptoms, minimally important differences (MIDs) have been established for many PROs. MIDs represent the smallest difference in symptom or functional scores that are noticeable for the patient. For the present study, we followed the suggestion by Musoro et al. to choose 10 points as the MID for C30 and CR29 scores for advanced colorectal cancer patients (Musoro et al. 2020). Reference values were also used to compare the scores as recommended by the EORTC QLQ-C30 Scoring Manual (Fayers et al. 2001; Scott et al. 2008; Whistance et al. 2009).

We also examined the survival of patients within the study period between T0 and T1 by using the documented dates of death in the EDIUM study database.

### Statistical analysis

Descriptive results include relative and absolute frequencies with mean and standard deviations for T0 and T1. For all selected EORTC QLQ-C30 and -CR29 items, raw scores as well as transformed scores are reported. The participants’ characteristics are reported stratified by colorectal cancer localization. The dropout cohort between T0 and T1 was investigated to detect differences in participants’ characteristics and reported pain compared to the patients who completed the questionnaire at T1 (Appendix Tables S1 and S2). To investigate the correlation between quality of life and pain at T1, Pearson’s correlation coefficient was analysed. Logistic regression analysis was performed to examine the relationship between quality of life and pain at T0 and 1-year survival (adjusted for age, educational level, gender, insurance status). The model performance was examined using the AIC, BIC, and Tjur’s *R*^2^. A p value < 0.05 was interpreted as statistically significant. Analysis was performed using the R statistical software program, version 4.3.1, using the “gtsummary”, “ggsankey” and “performance” packages (Lüdecke et al. 2021; Sjoberg 2021; Sjoberg et al. 2020).

## Results

### Study characteristics

The sample for this analysis consisted of 147 palliatively treated colorectal cancer patients aged between 40 and 90 years. Eighty per cent of the patients were over the age of 60, and over 70% were male. Their mean ages were 65 (10) years in colon cancer patients and 68 (9) years in rectal cancer patients. In both groups, most study participants had a certificate from a lower secondary school as their highest school-leaving qualification (colon: 75%, rectum: 78%). Details of the study cohort characteristics are shown in Table 1.

**Table 1:** Characteristics of the study cohort

Characteristics	Colon cancer patients (*n* = 66)	Rectal cancer patients (*n* = 81)
Age ^*1, 2*^	65 (10)	68 (9)
40–49	3 (4.5%)	2 (2.5%)
50–59	15 (23%)	8 (9.9%)
60–69	25 (38%)	34 (42%)
70–79	17 (26%)	31 (38%)
> 79	6 (9.1%)	6 (7.4%)
Gender ^*2*^		
Female	17 (26%)	17 (21%)
Male	49 (74%)	64 (79%)
Highest school education ^*2*^		
Higher secondary school	13 (21%)	13 (17%)
Lower secondary school	46 (75%)	60 (78%)
None	1 (1.6%)	1 (1.3%)
Other	1 (1.6%)	3 (3.9%)
Unknown	5	4
Insurance status ^*2*^		
Statutory health insurance	55 (89%)	67 (88%)
Private health insurance	6 (9.7%)	7 (9.2%)
Other/none	1 (1.6%)	2 (2.6%)
Unknown	4	5

^*1*^ Mean (SD); ^*2*^ n (%)

**Table 2:** EORTC QLQ-C30 and -CR29: quality of life and pain levels at T0 and T1. The pain score consists of the two items “interference with daily activities, last week” and “pain, last week”. The quality of life score consists of the two items “global health status” and “overall quality of life”. The scores for abdominal pain, buttock pain, and dysuria are converted from the corresponding items. A higher value for the pain scores indicates more severe pain, and a higher value for the quality of life score indicates a better quality of life status (both ranging from 0 to 100)

	Colon cancer patients (*n* = 66)		Rectal cancer patients (*n* = 81)	
	T0	T1	T0	T1
Quality of life^1, *^	51 (25)	56 (22)	52 (24)	51 (22)
Unknown	0	0	1	0
Pain^1, *^	34 (33)	35 (32)	26 (32)	35 (32)
Unknown	0	0	0	0
Pain, last week^2^				
Not at all	23 (34.85%)	25 (37.88%)	42 (51.85%)	29 (35.80%)
A little	20 (30.30%)	20 (30.30%)	17 (20.99%)	29 (35.80%)
Quite a bit	14 (21.21%)	15 (22.73%)	16 (19.75%)	15 (18.52%)
Very much	9 (13.64%)	6 (9.09%)	6 (7.41%)	8 (9.88%)
Unknown	0	0	0	0
Interference with daily activities, last week^2^				
Not at all	32 (50.79%)	24 (37.50%)	48 (60.76%)	31 (38.27%)
A little	11 (17.46%)	18 (28.13%)	12 (15.19%)	26 (32.10%)
Quite a bit	14 (22.22%)	13 (20.31%)	11 (13.92%)	13 (16.05%)
Very much	6 (9.52%)	9 (14.06%)	8 (10.13%)	11 (13.58%)
Unknown	3	2	2	0
Abdominal pain^1, *^	33 (34)	29 (30)	21 (28)	18 (24)
Unknown	0	0	0	0
Abdominal pain, last week^2^				
Not at all	27 (40.91%)	28 (42.42%)	46 (56.79%)	46 (56.79%)
A little	20 (30.30%)	21 (31.82%)	21 (25.93%)	27 (33.33%)
Quite a bit	12 (18.18%)	14 (21.21%)	12 (14.81%)	7 (8.64%)
Very much	7 (10.61%)	3 (4.55%)	2 (2.47%)	1 (1.23%)
Unknown	0	0	0	0
Buttock pain^1, *^	10 (23)	19 (28)	33 (36)	24 (32)
Unknown	1	0	1	0
Buttock pain, last week^2^				
Not at all	52 (80%)	41 (62.12%)	38 (47.50%)	47 (58.02%)
A little	7 (10.77%)	14 (21.21%)	15 (18.75%)	14 (17.28%)
Quite a bit	5 (7.69%)	9 (13.64%)	18 (22.50%)	16 (19.75%)
Very much	1 (1.54%)	2 (3.03%)	9 (11.25%)	4 (4.94%)
Unknown	1	0	1	0
Dysuria^1, *^	4 (13)	4 (12)	4 (13)	8 (19)
Unknown	1	0	0	1
Dysuria, last week^2^				
Not at all	58 (89.23%)	59 (89.39%)	73 (90.12%)	66 (82.50%)
A little	6 (9.23%)	6 (9.09%)	6 (7.41%)	10 (12.50%)
Quite a bit	1 (1.54%)	1 (1.52%)	2 (2.47%)	3 (3.75%)
Very much	0 (0%)	0 (0%)	0 (0%)	1 (1.25%)
Unknown	1	0	0	1

^1^ Mean (SD); ^2^ n (%); * converted score

### Pain and quality of life

The descriptive results show different trends in pain levels for colon and rectal cancer patients. Rectal cancer patients had higher “buttock pain” levels (mean: 33 (SD 36)) than colon patients at T0, which decreased at T1 (mean: 24 (SD: 32)). In contrast, “buttock pain” levels among colon cancer patients showed an increase at T1: the mean for “buttock pain” in colon cancer patients increased from 10 (SD: 23) to 19 (SD: 28). “Abdominal pain” decreased in both rectal and colon cancer patients. The mean for “dysuria” increased slightly in rectal cancer patients (T0: 4 (SD 13), T1: 8 (SD 19)), but remained stable in colon cancer patients. The overall “pain” score showed a higher increase in rectal cancer patients (T0: 26 (32), T1: 35 (32)) than in colon cancer patients (T0: 34 (33), T1: 35 (32)) (Table 2).

The overall quality of life levels remained stable in both groups. Analyses showed a moderate negative correlation between pain and quality of life both at T0 (*r* (144) = − 0.48, *p* < 0.001) and at T1 (r (145) = − 0.49, *p* < 0.001).

Comparison with the QLQ-C30 and -CR29 reference values showed that the study participants’ quality of life scores were below the reference value (cut-off: 61) for both patient groups at both measurement times. At T0 and T1, the mean total “pain” score was above the cut-off (24) for both groups. Changes in pain and quality of life at T1 were below the MID of 10 points (Musoro et al. 2020).

**Fig. 2:**
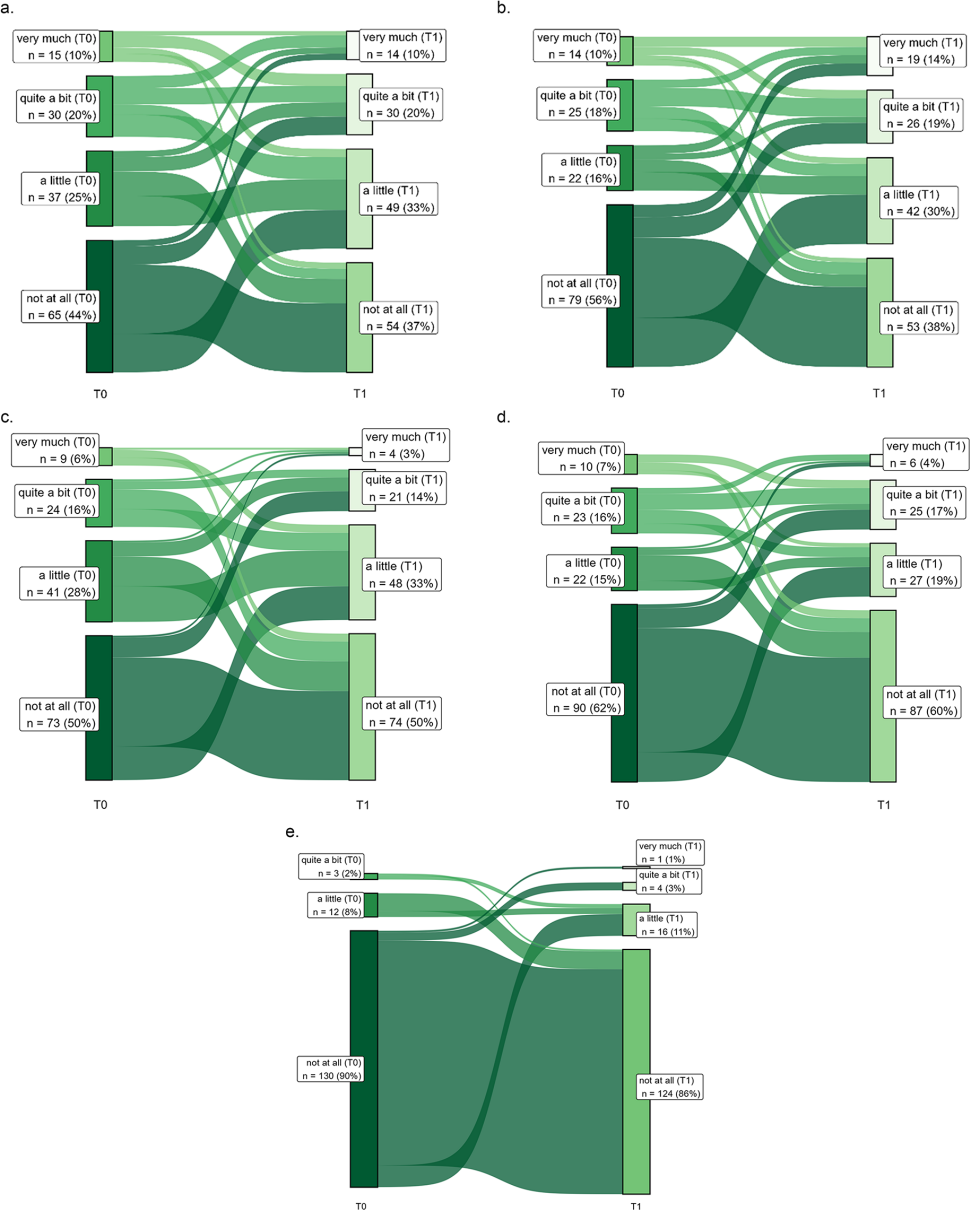
Sankey diagram for pain items from the EORTC QLQ-CR29 and C30 in colorectal cancer patients at T0 and T1. a, Pain (*n* = 147); b, interference with daily activities (*n* = 140); c, abdominal pain (*n* = 147); d, buttock pain (*n* = 145); e, dysuria (*n* = 145). The Sankey diagrams only include colorectal cancer patients who responded to the item at T0 and T1

Figure 2 shows the development of pain symptoms for T0 and T1 for the items “pain”, “interference with daily activities”, “abdominal pain”, “buttock pain”, and “dysuria”, in Sankey plots. “Interference with daily activities” showed the highest increase: 49.2% of colon cancer patients reported having a little or more than a little interference with daily activities due to pain at T0, increasing to 62.5% at T1. In rectal cancer patients, the interference of pain with their daily activities showed an increase from 39.24 to 61.73% at T1 (Fig. 2).

To examine the differences between the patients who were able to complete the T1 questionnaires and those who died within 12 months (*n* = 186), logistic regression analysis was conducted, with 1-year survival (yes vs. no) as the dichotomized outcome. The logistic regression for overall quality of life (T0) and survival showed a relationship indicating higher odds of 1-year survival with a higher quality of life (T0) (adjusted OR = 0.98, *p* < 0.01, Tjur’s *R*^2^ = 0.16). For pain (T0) as the predictor, logistic regression also indicated higher odds of 1-year survival with lower pain levels (T0) (adjusted OR = 1.01, *p* < 0.01, Tjur’s *R*^2^ = 0.16). Details of the logistic regression are shown in Table S3.

### Dropout analysis

The results of the dropout analysis showed that patients who did not complete the questionnaire 12 months after treatment initiation, although still alive, did not differ significantly from those who answered the T1 questionnaire regarding participants’ characteristics, or in pain levels at T0 (Appendix Tables S1 and S2).

## Discussion

Overall, these results highlight the symptomatic burden of pain that palliatively treated colorectal cancer patients face before and 12 months after the initiation of treatment. Pain at T0 predicted survival at T1, but the results need to be interpreted with caution due to the smallness of the sample and the lack of potential confounding variables. It also remains unclear whether the reported pain is caused by cancer symptoms, treatment, or other comorbidities.

Some of the descriptive findings deserve highlighting. The highest increase can be seen in the reporting of “interference with daily activities due to pain” for both colon and rectal cancer patients. Although there were changes in the individual pain items in both groups, “overall quality of life” did not differ substantially in either group. Taking the published reference MIDs into account (Musoro et al. 2020), the changes in pain and quality of life scores between T0 and T1 were not clinically relevant, but the reference values for “pain” were exceeded in the study population and reduced for “overall quality of life”.

The study results illustrate the very substantial burden of pain for palliatively treated colorectal cancer patients, which affects patients’ quality of life, as previous research has shown (Rodriguez et al. 2019). The results of the correlation analysis are consistent with previous research showing a moderate negative correlation between pain and quality of life, indicating that lower pain levels are associated with a higher quality of life at both time points. These results are in line with the results of the meta-analysis by Flyum et al. (Flyum et al. 2021). Sociodemographic as well as clinical variables were associated with the health-related quality of life, indicating the importance of tailoring a treatment plan to the patients’ symptoms (Flyum et al. 2021). As part of the present study, we evaluated the stoma status in rectal cancer patients along with their pain symptoms. The analysis did not show any statistically significant differences between patients with and without a stoma at T1 (results available on request).

Forty-four per cent of the palliatively treated patients who had completed the T0 questionnaire and for whom death was documented passed away within the 1-year follow-up period. The presented findings are in line with known colorectal cancer epidemiology (Robert Koch-Institut 2023; Wilson et al. 2023). In addition, the results of the dropout analysis, which analysed the study participants’ characteristics and pain levels, did not show any significant differences between patients who completed the T1 questionnaire and those who did not.

The results of the logistic regression analysis show a small relationship between pain/overall quality of life and survival. With adjustment of the models for relevant confounders, thus improving the goodness of fit, however, the relatively low *R*^2^ suggests that there are many other factors besides pain or quality of life that might explain the variation in survival.

To the best of our knowledge, this is one of the first studies to investigate pain specifically in palliatively treated colorectal cancer patients. The detailed results for surgically treated colorectal cancer patients published by Kowalski et al. showed that they had a lower mean “pain” score than the palliative subgroup presented here (Kowalski et al. 2022).

The management of pain in palliative care is a complex challenge that requires interdisciplinary approaches, as has already been stated for other end-stage diseases (Raina et al. 2018). It is important to monitor pain symptoms regularly to flexibly adapt to the patient’s pain status. In view of the wide range of pain types investigated by the present study and the fact that a high symptom burden was observed for all types of pain, the results confirm the need for an interdisciplinary approach. Palliative care for cancer patients should be tailored to the needs of the patient– by evaluating PROs in palliative cancer patients, for instance, as proposed by a recent commentary on the American Society of Clinical Oncology update on palliative care for cancer patients (Crowley et al. 2024).

### Strengths and limitations

The EDIUM study is being conducted in Colorectal Cancer Centres in Germany, Austria and Switzerland that are certified by the German Cancer Society. Certified centres meet a number of quality requirements and have better oncological outcomes on average than uncertified units (Schmitt et al. 2023). The documentation of clinical characteristics across certified centres is standardized and reviewed annually for certification purposes, ensuring data validity and reliability. However, the results may not be generalizable beyond certified centres. In addition, the use of scores and cut-off values in this study may not fully capture the patients’ pain status. The thresholds used are established based on population averages but may not account for variability in the population.

A recent review by Hasson et al. (Hasson et al. 2020) identifies research gaps in the field of palliative care and in particular highlights how difficult the recruitment of palliatively treated cancer patients for research generally is. For EDIUM as well, the centres reported having difficulties in including these patients in the study. This may limit the representativeness of the study population. For example, it might explain the age difference between the study population presented here and the overall colorectal cancer population in Germany. The younger mean age in the study population may perhaps be a result of the willingness of severely ill cancer patients to participate in studies while already receiving palliative care. Since an older palliatively treated population would be expected to be even more burdened by pain, the study may still underestimate the true burden and needs of these patients (Finnerty et al. 2019). A further limitation is the lack of information on the sites of metastases, pain medication, or other treatments like radiotherapy administered outside the certified centre, potentially affecting the patient’s pain trajectory.

## Conclusion

The results presented here show high levels of pain among colorectal cancer patients 12 months after the initiation of treatment, with low quality of life values. Yet symptom control and an improved quality of life, as defined in the palliative care guidelines, do not appear to be achieved. To follow oncologic guidelines for palliatively treated cancer patients, adequate symptom management is needed to improve the patients’ quality of life. To make it possible to provide adequate symptom-specific care and improve quality of life for patients with higher-stage cancer, symptom relief, including pain, should be monitored more closely. To improve the care of palliatively treated colorectal cancer patients, tailored care that considers the individual needs of cancer patients is crucial. Future research needs to focus on specific needs for adequate pain management, taking into account palliative treatment options and possible other factors such as clinical variables and patient characteristics.

## Electronic supplementary material

Below is the link to the electronic supplementary material.

Supplementary Material 1

Supplementary Material 2

Supplementary Material 3

## Data Availability

The data that support the findings of this study are not openly available due to data policy framework of the EDIUM study. Aggregated data can be requested from the corresponding author.
