# Correction: Pain and overall quality of life in palliatively treated colorectal cancer patients 1 year after diagnosis– results from the EDIUM cohort

**DOI:** 10.1007/s00432-025-06399-0

**Published:** 2026-02-03

**Authors:** Sophie Klara Schellack, Clara Breidenbach, Christoph Kowalski, Ulrich Wedding, Birgitt van Oorschot, Thomas Seufferlein, Stefan Benz, Martin Schnell, Jörg Köninger, Christina Klein, Johann Ockenga, Björn Freitag, Uwe A. Wittel, Roger Wahba, Mia Kim, Saleem Elhabash, Pompiliu Piso, Dirk Weyhe, Jörg Bunse, Maren Riechmann, Marco von Strauss, Sebastian Petzoldt, Philipp-Alexander Neumann, Vanessa Kolb, Nora Tabea Sibert

**Affiliations:** 1 German Cancer Society, Kuno-Fischer-Straße 8, 14057 Berlin, Germany; 2 University Medicine Jena, Kastanienstraße 1, 07747 Jena, Germany; 3 University of Würzburg, Josef-Schneider-Straße 11, 97080 Würzburg, Germany; 4 University Medicine Ulm, Albert-Einstein-Allee 23, 89081 Ulm, Germany; 5 Klinikum Sindelfingen-Böblingen, Calwer Straße 68, 71034 Böblingen, Germany; 6 Hegau-Bodensee-Klinikum Singen, Virchowstr. 10, 78224 Singen, Germany; 7 Klinikum Stuttgart, Kriegsbergstraße 60, 70174 Stuttgart, Germany; 8 Helios Park-Klinikum Leipzig, Strümpellstraße 41, 04289 Leipzig, Germany; 9 Bremen Klinikum Bremen-Mitte, Sankt-Jürgen-Str. 1, 28205 Bremen, Germany; 10 St. Josef-Hospital, Gudrunstraße 56, 44791 Bochum, Germany; 11 Kliniken Nordoberpfalz– Klinikum Weiden, Söllnerstraße 16, 92637 Weiden in der Oberpfalz, Germany; 12 Helios Klinikum Berlin-Buch, Schwanebecker Chaussee 50, 13125 Berlin, Germany; 13 München Klinik Neuperlach, Sanatoriumspl. 2, 81545 München, Germany; 14 Johannes Wesling Klinikum Minden, Hans-Nolte-Straße 1, 32429 Minden, Germany; 15 Barmherzige Brüder Regensburg, Prüfeninger Str. 86, 93049 Regensburg, Germany; 16 Pius Hospital University Medicine Oldenburg, Georgstraße 12, 26121 Oldenburg, Germany; 17 Sana Klinikum Lichtenberg, Fanningerstraße 32, 10365 Berlin-Lichtenberg, Germany; 18 Sana Klinikum Hof, Hochfranken, Eppenreuther Straße 9, 95032 Hof, Germany; 19 St. Claraspital Basel, Kleinriehenstrasse 30, 4058 Basel, Switzerland; 20 DRK Kliniken Berlin-Treptow– Köpenick, Salvador- Allende-Str. 2– 8, 12559 Berlin, Germany; 21 Klinikum Rechts der Isar, TUM University Hospital, TU Munich, Ismaninger Str. 22, Munich, Germany; 22 OnkoZert GmbH, Gartenstraße 24, Neu-Ulm, Germany; 23 Department of Gynaecology and Obstetrics, CIO ABCD, Oncological Health Services Research with a Focus on Digital Medicine, University Hospital Düsseldorf, Heinrich-Heine University Düsseldorf, Moorenstraße 5, Düsseldorf, Germany

**Correction to: Journal of Cancer Research and Clinical Oncology (2025) 151:127** 10.1007/s00432-025-06186-x

Following the publication of the article, errors were discovered in Table 2, Figure 2, and Table S2. Additionally, the text in the main body of the article related to the affected areas also requires correction.

In the abstract section of this article, the sentence was incorrectly given as “Higher pain levels persisted at both time points, with no patients reporting absence of pain.” and should have been “Higher pain levels persisted at both time points”

In the Pain and quality of life section of this article, the paragraph was incorrectly given as “Figure 2 shows the development of pain symptoms for T0 and T1 for the items “pain”, “interference with daily activities”, “abdominal pain”, “buttock pain”, and “dysuria”, in Sankey plots. None of the colorectal cancer patients reported having no pain at all at either of the two time points. “Interference with daily activities” showed the highest increase: 44% of colon cancer patients reported having more than a little interference with daily activities due to pain at T0, increasing to 57% at T1. In rectal cancer patients, the interference of pain with their daily activities showed an increase from 32 to 56% at T1 (Fig. 2).” and should have been “Figure 2 shows the development of pain symptoms for T0 and T1 for the items “pain”, “interference with daily activities”, “abdominal pain”, “buttock pain”, and “dysuria”, in Sankey plots. “Interference with daily activities” showed the highest increase: 49.2% of colon cancer patients reported having a little or more than a little interference with daily activities due to pain at T0, increasing to 62.5% at T1. In rectal cancer patients, the interference of pain with their daily activities showed an increase from 39.24% to 61.73% at T1 (Fig. 2).”

In this article, the Figure 2 caption was incorrectly given as 'Sankey diagram for pain items from the EORTC QLQ-CR29 and C30 in colorectal cancer patients at T0 and T1. a, Pain (*n* = 120); b, interference with daily activities (*n* = 112); c, abdominal pain (*n* = 135); d, buttock pain (*n* = 129); e, dysuria (*n* = 144). The Sankey diagrams only include colorectal cancer patients who responded to the item at T0 and T1.' but should have been ' Sankey diagram for pain items from the EORTC QLQ-CR29 and C30 in colorectal cancer patients at T0 and T1. a, Pain (*n* = 147); b, interference with daily activities (*n* = 140); c, abdominal pain (*n* = 147); d, buttock pain (*n* = 145); e, dysuria (*n* = 145). The Sankey diagrams only include colorectal cancer patients who responded to the item at T0 and T1'.

In the Pain and quality of life section of this article, the paragraph was incorrectly given as “Overall, these results highlight the symptomatic burden of pain that palliatively treated colorectal cancer patients face before and 12 months after the initiation of treatment. No patients reported that they were free of pain at either T0 or T1. Pain at T0 predicted survival at T1, but the results need to be interpreted with caution due to the smallness of the sample and the lack of potential confounding variables. It also remains unclear whether the reported pain is caused by cancer symptoms, treatment, or other comorbidities.” and should have been “Overall, these results highlight the symptomatic burden of pain that palliatively treated colorectal cancer patients face before and 12 months after the initiation of treatment. Pain at T0 predicted survival at T1, but the results need to be interpreted with caution due to the smallness of the sample and the lack of potential confounding variables. It also remains unclear whether the reported pain is caused by cancer symptoms, treatment, or other comorbidities.”

The wrong Supplementary file 2 was originally published with this article; it has now been replaced with the correct file.

In Table 2 of this article, the data in the column headed To & T1 were incorrectly published. For completeness and transparency, the old incorrect version and the corrected version of Table 2 are displayed below.

Incorrect Table:

**Table 2:** EORTC QLQ-C30 and -CR29: quality of life and pain levelsat T0 and T1. The pain score consists of the two items “interferencewith daily activities, last week” and “pain, last week”. The quality oflife score consists of the two items “global health status” and “overallquality of life. The scores for abdominal pain, buttock pain, and dysuriaare converted from the corresponding items. A higher value forthe pain scores indicates more severe pain, and a higher value for thequality of life score indicates a better quality of life status (both rangingfrom 0 to 100)

	Colon cancer patients (n = 66)		Rectal cancer patients (n = 81)	
	T0	T1	T0	T1
Quality of life^1,*^	51 (25)	56 (22)	52 (24)	51 (22)
Unknown	0		1	
Pain^1,*^	34 (33)	35 (32)	26 (32)	35 (32)
Pain, last week^2^				
Not at all	0	0	0	0
A little	23 (40%)	25 (42%)	42 (56%)	29 (40%)
Quite a bit	20 (35%)	20 (33%)	17 (23%)	29 (40%)
Very much	14 (25%)	15 (25%)	16 (21%)	15 (21%)
Unknown	9	6	6	8
Interference with daily activities, last week^2^				
Not at all	0	0	0	0
A little	32 (56%)	24 (44%)	48 (68%)	31 (44%)
Quite a bit	11 (19%)	18 (33%)	12 (17%)	26 (37%)
Very much	14 (25%)	13 (24%)	11 (15%)	13 (9%)
Unknown	9	11	10	11
Abdominal pain^1,*^	33 (34)	29 (30)	21 (28)	18 (24)
Abdominal pain, last week^2^				
Not at all	0	0	0	0
A little	27 (46%)	28 (44%)	46 (58%)	46 (58%)
Quite a bit	20 (34%)	21 (33%)	21 (27%)	27 (34%)
Very much	12 (20%)	14 (22%)	12 (15%)	7 (8.8%)
Unknown	7	3	2	1
Buttock pain^1,*^	10 (23)	19 (28)	33 (36)	24 (32)
Unknown	1	0	1	0
Buttock pain, last week^2^				
Not at all	0	0	0	0
A little	52 (81%)	41 (64%)	38 (54%)	47 (62%)
Quite a bit	7 (11%)	14 (22%)	15 (21%)	14 (18%)
Very much	5 (7.8%)	9 (14%)	18 (25%)	16 (21%)
Unknown	2	2	10	4
Dysuria^1,*^	4 (13)	4 (12)	4 (13)	8 (19)
Unknown	1	0	0	1
Dysuria, last week^2^				
Not at all	0	0	0	0
A little	58 (89%)	59 (89%)	73 (90%)	66 (84%)
Quite a bit	6 (9.2%)	6 (9.1%)	6 (7.4%)	10 (13%)
Very much	1 (1.5%)	1 (1.5%)	2 (2.5%)	3 (3.8%)
Unknown	1	0	0	2

^1^Mean (SD); ^2^n (%); * converted score

Corrected Table:

**Table 2:** EORTC QLQ-C30 and -CR29: quality of life and pain levels at T0 and T1. The pain score consists of the two items “Interference with daily activities, last week” and “Pain, last week”. The quality of life score consists of the two items “global health status” and “overall quality of life”. The scores for abdominal pain, buttock pain, and dysuria are converted from the corresponding items. A higher value for the pain scores indicates more severe pain, and a higher value for the quality of life score indicates a better quality of life status (both ranging from 0 to 100)

	Colon cancer patients (n = 66)		Rectal cancer patients (n = 81)	
	T0	T1	T0	T1
Quality of life^1,*^	51 (25)	56 (22)	52 (24)	51 (22)
Unknown	0	0	1	0
Pain^1,*^	34 (33)	35 (32)	26 (32)	35 (32)
Unknown	0	0	0	0
Pain, last week^2^				
Not at all	23 (34.85%)	25 (37.88%)	42 (51.85%)	29 (35.80%)
A little	20 (30.30%)	20 (30.30%)	17 (20.99%)	29 (35.80%)
Quite a bit	14 (21.21%)	15 (22.73%)	16 (19.75%)	15 (18.52%)
Very much	9 (13.64%)	6 (9.09%)	6 (7.41%)	8 (9.88%)
Unknown	0	0	0	0
Interference with daily activities, last week^2^				
Not at all	32 (50.79%)	24 (37.50%)	48 (60.76%)	31 (38.27%)
A little	11 (17.46%)	18 (28.13%)	12 (15.19%)	26 (32.10%)
Quite a bit	14 (22.22%)	13 (20.31%)	11 (13.92%)	13 (16.05%)
Very much	6 (9.52%)	9 (14.06%)	8 (10.13%)	11 (13.58%)
Unknown	3	2	2	0
Abdominal pain^1,*^	33 (34)	29 (30)	21 (28)	18 (24)
Unknown	0	0	0	0
Abdominal pain, last week^2^				
Not at all	27 (40.91%)	28 (42.42%)	46 (56.79%)	46 (56.79%)
A little	20 (30.30%)	21 (31.82%)	21 (25.93%)	27 (33.33%)
Quite a bit	12 (18.18%)	14 (21.21%)	12 (14.81%)	7 (8.64%)
Very much	7 (10.61%)	3 (4.55%)	2 (2.47%)	1 (1.23%)
Unknown	0	0	0	0
Buttock pain ^1, *^	10 (23)	19 (28)	33 (36)	24 (32)
Unknown	1	0	1	0
Buttock pain, last week^2^				
Not at all	52 (80%)	41 (62.12%)	38 (47.50%)	47 (58.02%)
A little	7 (10.77%)	14 (21.21%)	15 (18.75%)	14 (17.28%)
Quite a bit	5 (7.69%)	9 (13.64%)	18 (22.50%)	16 (19.75%)
Very much	1 (1.54%)	2 (3.03%)	9 (11.25%)	4 (4.94%)
Unknown	1	0	1	0
Dysuria ^1, *^	4 (13)	4 (12)	4 (13)	8 (19)
Unknown	1	0	0	1
Dysuria, last week^2^				
Not at all	58 (89.23%)	59 (89.39%)	73 (90.12%)	66 (82.50%)
A little	6 (9.23%)	6 (9.09%)	6 (7.41%)	10 (12.50%)
Quite a bit	1 (1.54%)	1 (1.52%)	2 (2.47%)	3 (3.75%)
Very much	0 (0%)	0 (0%)	0 (0%)	1 (1.25%)
Unknown	1	0	0	1

^1^Mean (SD); ^2^n (%); * converted score

In this article, Fig. 2 appeared incorrectly and has now been corrected in the original publication. For completeness and transparency, the old incorrect version and the corrected version of Fig. 2 are displayed below.

Incorrect version of Fig. 2:

**Fig. 2:**
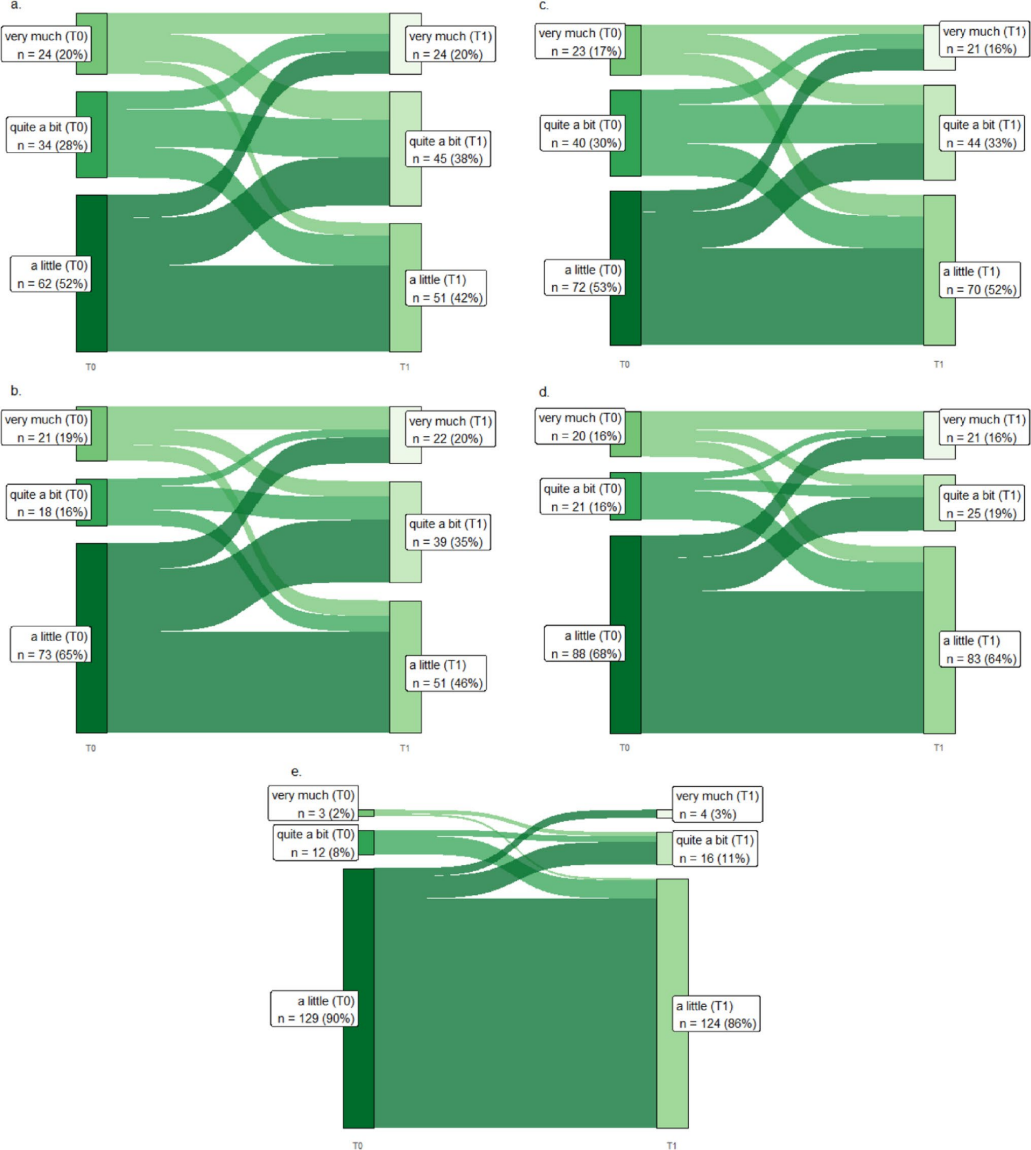
Sankey diagram for pain items from the EORTC QLQ-CR29 and C30 in colorectal cancer patients at T0 and T1. a, Pain (*n* = 120); b, interference with daily activities (*n* = 112); c, abdominal pain (*n* = 135); d, buttock pain (*n* = 129); e, dysuria (*n* = 144). The Sankey diagrams only include colorectal cancer patients who responded to the item at T0 and T1.

Corrected version of Fig. 2:

**Fig. 2:**
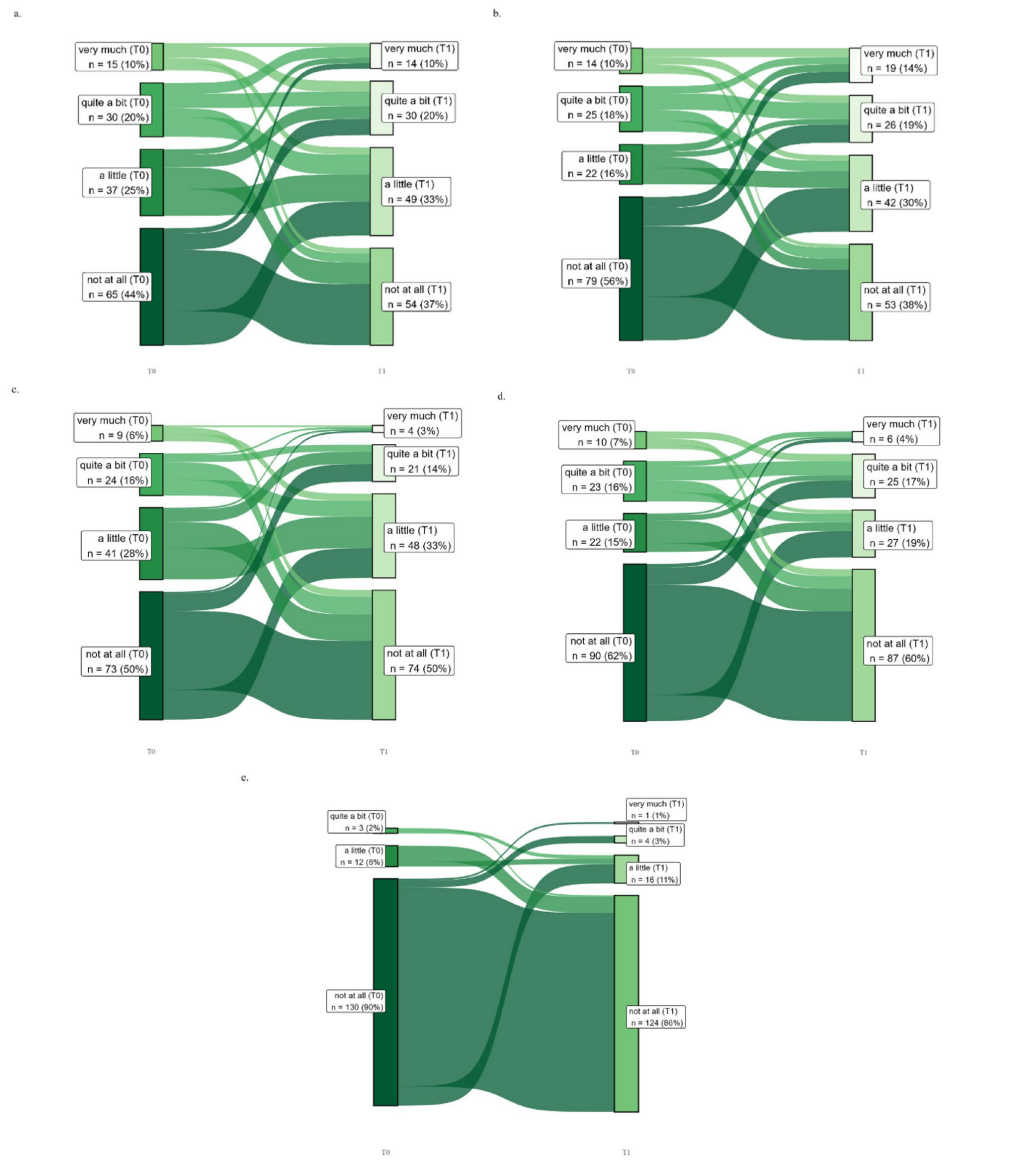
Sankey diagram for pain items from the EORTC QLQ-CR29 and C30 in colorectal cancer patients at T0 and T1. a, Pain (*n* = 147); b, interference with daily activities (*n* = 140); c, abdominal pain (*n* = 147); d, buttock pain (*n* = 145); e, dysuria (*n* = 145). The Sankey diagrams only include colorectal cancer patients who responded to the item at T0 and T1

The original article has been updated.

## Supplementary Information

Below is the link to the electronic supplementary material.

Supplementary Material 1

