# Supplementary material for: Pain and overall quality of life in palliatively treated colorectal cancer patients 1 year after diagnosis– results from the EDIUM cohort
Source: J Cancer Res Clin Oncol. 2025 Mar 31;151(4):127. doi: 10.1007/s00432-025-06186-x (PMC11958386; doi:10.1007/s00432-025-06186-x)
Supplement: Supplementary file 1 — Supplementary Material 1 [file 432_2025_6186_MOESM1_ESM.docx]

**Table S1**

Comparison of study participants’ characteristics with and without T1 questionnaire after 12 months. Study participants who died within the study period are excluded.

| Characteristic | Without T1 questionnaire (n = 97) | With T1 questionnaire (n = 147) |
| --- | --- | --- |
| Age *^1,2^* | 66 (13) | 66 (9) |
| < 40 | 2 (2.1%) | 0 (0%) |
| 40–49 | 9 (9.3%) | 5 (3.4%) |
| 50–59 | 19 (20%) | 23 (16%) |
| 60–69 | 30 (31%) | 59 (40%) |
| 70–79 | 19 (20%) | 48 (33%) |
| > 79 | 18 (19%) | 12 (8.2%) |
| Highest school education *^2^* |  |  |
| Higher secondary school | 20 (25%) | 26 (19%) |
| Lower secondary school | 59(73%) | 106 (77%) |
| None | 2 (2.5%) | 2 (1.4%) |
| Other | 0(0%) | 4 (2.9%) |
| Unknown | 16 | 9 |
| Gender *^2^* |  |  |
| Female | 30 (31%) | 34 (23%) |
| Male | 67 (69%) | 113 (77%) |
| Insurance status *^2^* |  |  |
| Statutory health insurance | 70 (85%) | 122 (88%) |
| Private health insurance | 11 (13%) | 13 (9.4%) |
| Other/none | 1 (1.2%) | 3 (2.2%) |
| Unknown | 15 | 9 |
| Localization *^2^* |  |  |
| Colon | 38 (39%) | 66 (45%) |
| Rectum | 59 (61%) | 81 (55%) |
| *^1^* Mean (SD); *^2^* n (%). | | |
