# Supplementary material for: Pain and overall quality of life in palliatively treated colorectal cancer patients 1 year after diagnosis– results from the EDIUM cohort
Source: J Cancer Res Clin Oncol. 2025 Mar 31;151(4):127. doi: 10.1007/s00432-025-06186-x (PMC11958386; doi:10.1007/s00432-025-06186-x)
Supplement: Supplementary file 2 — Supplementary Material 2 [file 432_2025_6186_MOESM2_ESM.docx]

**Table S2**

Comparison of self-reported pain in colorectal cancer patients at T0, with and without a T1 questionnaire. The pain score consists of the two items “Interference with daily activities, last week” and “Pain, last week”. The scores for abdominal pain, buttock pain, and dysuria are converted from the corresponding items.

| Characteristic | Without T1 questionnaire (n = 97) | With T1 questionnaire (n = 147) |
| --- | --- | --- |
| Quality of life (T0) *^1, *^* | 50 (27) | 51 (24) |
| Unknown | 2 | 1 |
| Pain (T0) *^1,^* * | 34 (33) | 29 (32) |
| Unknown | 1 | 0 |
| Pain, last week (T0) *^2^* |  |  |
| Not at all | 34 (35.42%) | 65 (44.22%) |
| A little | 31 (32.29%) | 37 (25.17%) |
| Quite a bit | 17 (17.71%) | 30 (20.41%) |
| Very much | 14 (14.58%) | 15 (10.20%) |
| Unknown | 1 | 0 |
| Interference with daily activities, last week (T0) *^2^* |  |  |
| Not at all | 44 (45.83%) | 80 (56.34%) |
| A little | 23 (23.96%) | 23 (16.20%) |
| Quite a bit | 19 (19.79%) | 25 (17.61%) |
| Very much | 10 (10.42%) | 14 (9.86%) |
| Unknown | 1 | 5 |
| Abdominal pain (T0) *^1,^* * | 31 (35) | 26 (31) |
| Unknown | 0 | 0 |
| Abdominal pain, last week (T0) *^2^* |  |  |
| Not at all | 45 (46.39%) | 73 (49.66%) |
| A little | 23 (23.71%) | 41 (27.89%) |
| Quite a bit | 19 (19.59%) | 24 (16.33%) |
| Very much | 10 (10.31%) | 9 (6.12%) |
| Unknown | 0 | 0 |
| Buttock pain (T0) *^1,^* * | 26 (36) | 23 (33) |
| Unknown | 1 | 2 |
| Buttock pain, last week (T0) *^2^* |  |  |
| Not at all | 55 (57.29%) | 90 (62.07%) |
| A little | 17 (17.71%) | 22 (15.17%) |
| Quite a bit | 13 (13.54%) | 23 (15.86%) |
| Very much | 11 (11.46%) | 10 (6.90%) |
| Unknown | 1 | 2 |
| Dysuria (T0) *^1,^* * | 7 (19) | 4 (13) |
| Unknown | 0 | 1 |
| Dysuria, last week (T0) *^2^* |  |  |
| Not at all | 84 (86.60%) | 131 (89.73%) |
| A little | 7 (7.22%) | 12 (8.22%) |
| Quite a bit | 5 (5.15%) | 3 (2.05%) |
| Very much | 1 (1.03%) | 0 (0%) |
| Unknown | 0 | 1 |
| *^1^* Mean (SD), *^2^* n (%); * transformed scores. | | |
