# Supplementary material for: Pain and overall quality of life in palliatively treated colorectal cancer patients 1 year after diagnosis– results from the EDIUM cohort
Source: J Cancer Res Clin Oncol. 2025 Mar 31;151(4):127. doi: 10.1007/s00432-025-06186-x (PMC11958386; doi:10.1007/s00432-025-06186-x)
Supplement: Supplementary file 3 — Supplementary Material 3 [file 432_2025_6186_MOESM3_ESM.docx]

**Table S3**

Results of logistic regression. The scale for pain and quality of life ranges from 0 to 100, with higher values indicating higher pain levels and a better quality of life.

| Effect | n | OR | 95% CI | p value | Tjur’s *R*^2^ | AIC | BIC |
| --- | --- | --- | --- | --- | --- | --- | --- |
| Quality of life at T0 | 356 | 0.98 | 0.97–0.99 | < 0.01 | 0.05 | 479.97 | 487.72 |
| Quality of life at T0 (adjusted) *^1^* | 313 | 0.98 | 0.97–0.99 | < 0.01 | 0.16 | 398.857 | 432.57 |
| Pain at T0 | 360 | 1.01 | 1.00–1.02 | < 0.01 | 0.03 | 492.24 | 500.01 |
| Pain at T0 (adjusted) *^1^* | 316 | 1.01 | 1.00–1.02 | < 0.01 | 0.16 | 400.05 | 433.85 |
| ^1^ Adjusted for highest school education, insurance status, age, gender.  AIC, Akaike information criterion; BIC, Bayes information criterion (BIC). | | | | | | | |
