# Supplementary material for: Pain and overall quality of life in palliatively treated colorectal cancer patients 1 year after diagnosis– results from the EDIUM cohort
Source: J Cancer Res Clin Oncol. 2025 Mar 31;151(4):127. doi: 10.1007/s00432-025-06186-x (PMC11958386; doi:10.1007/s00432-025-06186-x)
Supplement: Supplementary file 1 [file 432_2025_6186_MOESM2_ESM.docx]

**Table S2**

Comparison of self-reported pain in colorectal cancer patients at T0, with and without a T1 questionnaire. The pain score consists of the two items “Interference with daily activities, last week” and “Pain, last week”. The scores for abdominal pain, buttock pain, and dysuria are converted from the corresponding items.

| Characteristic | Without T1 questionnaire (n = 97) | With T1 questionnaire (n = 147) |
| --- | --- | --- |
| Quality of life (T0) ^*1, **^ | 50 (27) | 51 (24) |
| Unknown | 2 | 1 |
| Pain (T0) ^*1,*^ * | 34 (33) | 29 (32) |
| Unknown | 1 | 0 |
| Pain, last week (T0) ^*2*^ |  |  |
| A little | 34 (41%) | 65 (49%) |
| Quite a bit | 31 (38%) | 37 (28%) |
| Very much | 17 (21%) | 30 (23%) |
| Unknown | 15 | 15 |
| Interference with daily activities, last week (T0) ^*2*^ |  |  |
| A little | 44 (51%) | 80 (63%) |
| Quite a bit | 23 (27%) | 23 (18%) |
| Very much | 19 (22%) | 25 (20%) |
| Unknown | 11 | 19 |
| Abdominal pain (T0) ^*1,*^ * | 31 (35) | 26 (31) |
| Abdominal pain, last week (T0) ^*2*^ |  |  |
| A little | 45 (52%) | 73 (53%) |
| Quite a bit | 23 (26%) | 41 (30%) |
| Very much | 19 (22%) | 24 (17%) |
| Unknown | 10 | 9 |
| Buttock pain (T0) ^*1,*^ * | 26 (36) | 23 (33) |
| Unknown | 1 | 2 |
| Buttock pain, last week (T0) ^*2*^ |  |  |
| A little | 55 (65%) | 90 (67%) |
| Quite a bit | 17 (20%) | 22 (16%) |
| Very much | 13 (15%) | 23 (17%) |
| Unknown | 12 | 12 |
| Dysuria (T0) ^*1,*^ * | 7 (19) | 4 (13) |
| Unknown | 0 | 1 |
| Dysuria, last week (T0) ^*2*^ |  |  |
| A little | 84 (88%) | 131 (90%) |
| Quite a bit | 7 (7.3%) | 12 (8.2%) |
| Very much | 5 (5.2%) | 3 (2.1%) |
| Unknown | 1 | 1 |
| ^*1*^ Mean (SD), ^*2*^ n (%); * transformed scores. |  |  |
